# Case Report: Successful peripartum management of acquired hemophilia A relapse during pregnancy

**DOI:** 10.3389/fmed.2026.1963805

**Published:** 2026-10-27

**Authors:** Hongyun Chen, Xiaochen Yu, Tienan Zhu

**Affiliations:** 1 Department of Hematology, Peking Union Medical College Hospital, Chinese Academy of Medical Sciences & Peking Union Medical College, Beijing, China; 2 Department of Nephrology, Peking Union Medical College Hospital, Chinese Academy of Medical Sciences & Peking Union Medical College, Beijing, China

**Keywords:** acquired hemophilia a, case report, literature review, peripartum management, pregnancy

## Abstract

**Background:**

Few reports of Pregnancy-related Acquired hemophilia A (AHA) manifested prenatally. Due to rarity, there are no definitive guidelines for the peripartum management of pregnant women with AHA and their newborns.

**Case presentation:**

we report a 30-year-old woman with relapsed AHA during pregnancy. She maintained partial remission with cyclosporin, underwent cesarean section with rFVIIa prophylaxis, and controlled postpartum bleeding. The newborn had transient FVIII inhibitors without bleeding.

**Conclusion:**

Bypassing agents effectively prevent postpartum hemorrhage in AHA. Close monitoring is necessary for newborns. We observed that immunosuppressive therapy administered during pregnancy and the postpartum period failed to eliminate FVIII inhibitors, highlighting contraception necessity for non-remission patients.

## Introduction

Acquired hemophilia A (AHA) is a rare bleeding disorder caused by autoantibodies or inhibitors against coagulation factor VIII (1, 2). Approximately 50% of the cases are idiopathic, while the others may be secondary to autoimmune diseases, malignancies, dermatological conditions, infections, or pregnancy (3, 4). Pregnancy-related AHA, with a reported incidence of 2%–21% of the patients with AHA, often occurs postpartum, within days or months of the first pregnancy (3, 5, 6). However, very few cases of AHA manifesting prenatally were published. The optimal management throughout the pregnancy and delivery remains unambiguous. Besides, the newborns also suffer bleeding risks because FVIII inhibitors are primarily of IgG class which can cross the placenta into the fetus (7), underscoring the importance of close monitoring. Here, we report a case of a gravida with anrelapsed AHA relapse during pregnancy, in which an uneventful pregnancy and a successful delivery were achieved after the administration of optimal treatment regimens.

## Case report

In July 2018, a 30-year-old woman was admitted to our hospital, presenting with gross bleeding during a laparoscopic surgery for corpus luteum rupture. An isolated, obvious prolongation of Activated Partial Thromboplastin Time (APTT) of 119s was observed. Prothrombin time (PT), platelet count fell within reference ranges. A 1:1 incubated APTT mixing study (2-hour incubation at 37 ℃) showed timedependent non-correction of APTT, confirming the presence of a time-dependent circulating coagulation inhibitor. The FVIII activity was less than 1%, and the FVIII inhibitor titer was 992 BU/mL by Bethesda assay. Von Willebrand factor antigen was 94.2%. Tests for lupus anticoagulant and other antiphospholipid antibodies were negative. The patient had no personal or family history of abnormal bleeding and no manifestations of autoimmune diseases. Concurrent findings included positive Hepatitis B surface antigen (HBsAg), Hepatitis B e antigen (HBeAg), and Hepatitis B core antibody (HBcAb), with a Hepatitis B virus DNA level of 2.8 × 10^8^ copies/mL. We systematically ruled out common secondary etiologies: complete serological screening for connective-tissue autoimmune diseases (ANA, anti-dsDNA, anti-ENA spectrum, rheumatoid factor) were all negative; tumor markers, chest-abdomen-pelvis imaging excluded underlying malignancy; no history of drug exposure associated with AHA; no active bacterial infection. The patient received initial prednisone 40 mg(1 mg/kg) qd together with CTX 50/100 mg qod. After 1 month, prednisone was reduced to 35 mg qd. No decline in FVIII inhibitor or rise in FVIII activity was observed, suggestive of poor response. Rituximab was not administered initially owing to high HBV DNA levels. The immunosuppressant was switched to azathioprine (AZA) 100 mg qd with no benefit, then changed to Cyclosporin 75 mg qd but the responce was not satisfactory. After Rituximab (375 mg/m^2^ weekly  ×  4) was administrated, FVIII activity gradually increased to 33% and FVIII inhibitor was undetectable 6 months after administration, indicating a partial remission (8). Cyclosporin was chosen as maintenance therapy. Antiviral therapy with entecavir 0.5 mg qd was initiated in July 2018. Given unsatisfactory reduction in HBV DNA levels, tenofovir 300 mg qd was subsequently added. Following combination therapy, HBV DNA rapidly declined to below the lower limit of detection (Figure 1).

**Figure 1:**
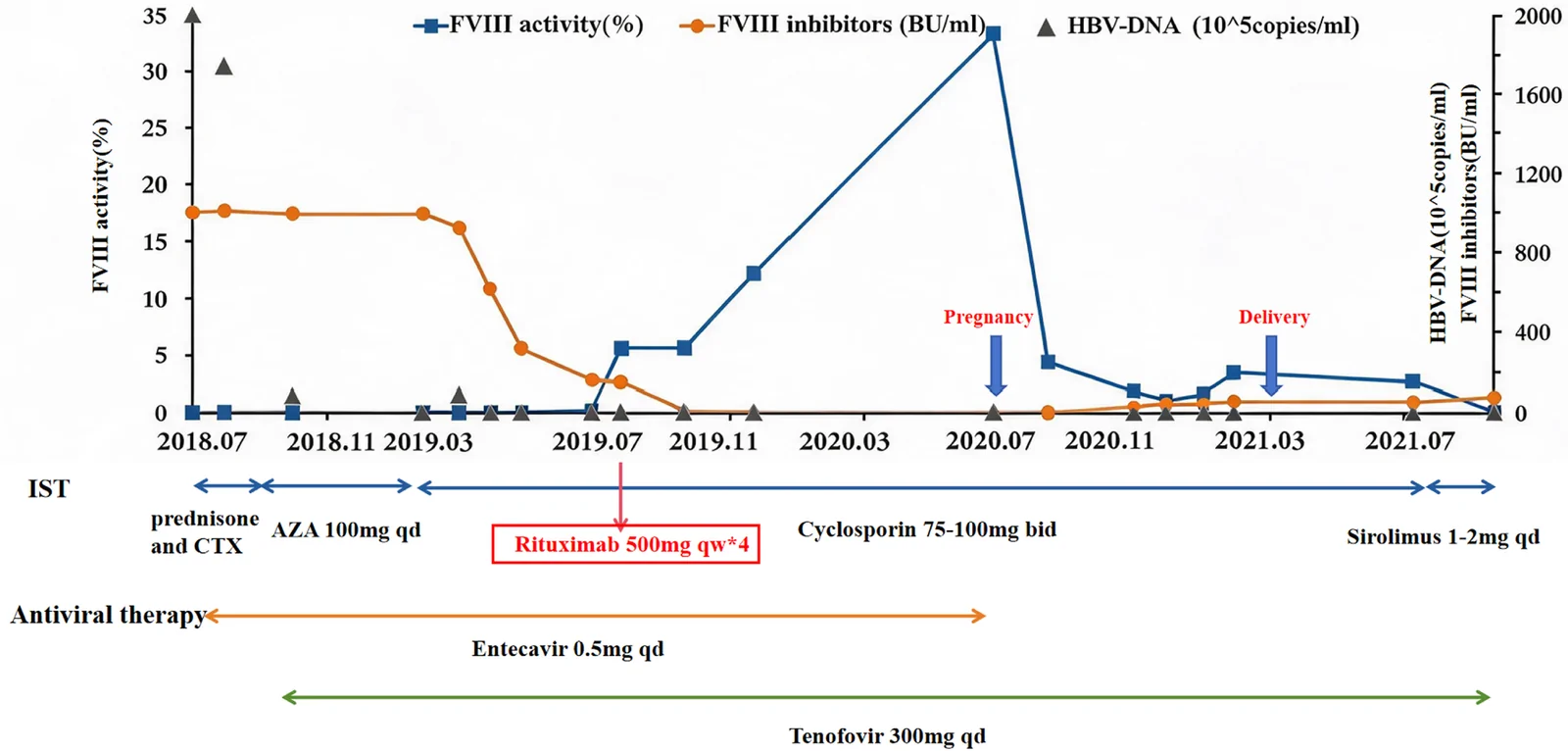
Serial laboratory dynamics and treatment trajactory during follow-up. AZA:azathioprine .

In July 2020, the patient sought medical attention due to an unintended pregnancy. After a comprehensive discussion about the risks of continued gestation, the patient decided to proceed with the pregnancy. Historical refractoriness to high-dose combined regimen corticosteroid makes clinical benefit uncertain even years later and the patient was unwilling to receive another course of corticosteroid treatment. She was advised to continue taking cyclosporin 75 mg bid. Regular monitoring indicated a gradual increase in FVIII inhibitor titers from 0 to 51.2 BU/mL, with a concurrent decrease of FVIII activity from 30% to 3.4% before delivery (Figure 1). Fortunately, pregnancy progressed without significant bleeding episodes, and routine prenatal screenings showed no fetal abnormalities. At 37 ^+^ ^4^ weeks, a cesarean section was performed, preceded by an immediate intravenous injection of recombinant activated Factor VII (rFVIIa) at a dose of 4 mg(88.8 μg/kg). A male baby with Apgar scores of 5 at 1 and 5 min, respectively, was born. Active bleeding at the incision site and cervix was noted. The hemorrhage ceased after immediate uterine artery embolization and uterine tamponade were performed. The operative blood loss was estimated to be 900 mL, with hemoglobin levels dropping from 110 g/L to 85 g/L. Afterwards, the patient was transfused with two units of red blood cell concentrates.400 mL FFP was transfused due to intra-operative evidence of mild global coagulation factor consumption after uterine artery embolization and tamponade for surgical bleeding. Intraoperative PT and INR were mildly prolonged, consistent with consumption of multiple coagulation factors rather than isolated FVIII neutralization by the inhibitor. The bypassing agent, rFVIIa, was repeatedly administered 4 h after surgery and then every 8 h. Due to financial issues, the hemostatic regimen was switched to Prothrombin Complex Concentrate (PCC) 900 U q12 h intravenously. PCC was discontinued on postoperative day 6 considering the condition was stable. Final checks showed an APTT of 87.5s, FVIII activity at 1.1%, and FVIII inhibitor at 44.8 BU/mL. As regards to the condition of the newborn, immediate umbilical cord blood testing showed a prolonged APTT of 120s. The FVIII activity was 1.2%, and the titer of the FVIII inhibitor was 11.2 BU/mL, indicating the presence of a maternal-derived FVIII inhibitor. Cranial and abdominal ultrasounds showed no bleeding signs. Thus, no specific hemostatic treatment was given. A close monitoring showed that the newborn's FVIII inhibitors disappeared on day 14 and he had no bleeding episodes.

## Follow-up and outcomes

After discharge, the patient continued cyclosporin 75 mg bid and FVIII activity increased to 2.7% and FVIII inhibitor increased to 52BU/mL. Sirolimus was attempted but the outcome was not satisfactory. Given that rituximab was effective, it was administrated again, but remission was not achieved. At latest follow-up, the child was 5 years old. He demonstrated age-appropriate growth and had experienced no bleeding events since spontaneous clearance of the transplacentally transferred FVIII inhibitor on postnatal day 14.

## Discussion

According to previous studies, pregnancy-associated AHA accounts for 2%–21% of AHA cases. Though pregnancy-related AHA typically occurs postpartum, it can also occur before delivery and present substantial management challenges (9–11).In our case, the primigravida was diagnosed with AHA occurring in the setting of chronic HBV infection, before her pregnancy. Partial remission was achieved after multiple immunosuppressants were given. The disease exacerbated after an accidental pregnancy, making it difficult to manage.

Due to the low occurrence, there are no widely accepted protocols for the peripartum treatment of AHA. We searched the published literature on AHA manifesting prenatally by PubMed and found only 13 cases. Table 1 gives a chronologic review of relevant literature. Although early eradication of inhibitors can facilitate disease remission and avoid potential bleeding complications in both mother and infant, the use of recommended treatments like cyclophosphamide and rituximab is discouraged during pregnancy due to teratogenic risks (12). Corticosteroids can be used during pregnancy but offer only about a 60% remission rate as monotherapy (13) and may increase the risk of complications such as gestational diabetes and osteoporosis. Cyclosporin and azathioprine are considered safe for use during pregnancy, but experience with these drugs in pregnancy-related AHA is limited (14). Intravenous immunoglobulin, even safe for use during pregnancy (15), does not have a definitive effect on AHA and is not currently recommended (3, 13). In our case, we didn't reintroduce prednisone for the patient was refractory to prior steroid therapy. The patient continued the cyclosporin regimen yet inhibitor levels continued to rise throughout pregnancy until delivery. No bleeding episode was observed during her pregnancy, which may be partially explained by hypercoagulation propensity. Bleeding control during and after the delivery is another tough issue to address. Bypassing agents were recommended in AHA hemostatic treatment. The application of rFVIIa is increasingly common in obstetric hemorrhage (16), and its short half-life can reduce the risk of thrombosis in the postpartum hypercoagulable state. Since the patient received a cesarean section, a prophylactic bypassing agent, rFVIIa, was administrated and given regularly to avoid postpartum hemorrhage. PCC was also given in our case. Hemostatic treatment was tapered and then stopped considering the risk for thrombosis. Unfortunately, unlike other pregnant-associated AHA patients, the FVIII inhibitor failed to decrease even after intensified IST.

**Table 1:** Cases of AHA diagnosed prenatally in the literature.

Case no.	author	Year of publishment	Age (year)	FVIII		Hemorrhagic signs in pregnancy	IST	Hemostatic treatment	Delivery mode	Postpartum bleeding episode	FVIII in newborn		Bleeding complications in neonate	Maternal prognosis
				Initial activity (%)	Inhibitor titers (BU/mL)						Initial activity (%)	Inhibitor titers (BU/mL)		
1 (19)	Frick	1953	25	NA	NA	Hematuriaecchymoses	NA	NA	Vaginal	Vaginal bleeding	NA	Detected	NA	Self-limited
2 (20)	Marengo	1972	14	3	14	Hematuria	NA	FVIII	Vaginal	Vaginal bleeding	80	None	NA	No remmission
3 (21)	Voke	1977	33	Undetectable	Detected	Spontaneous bruises	NA	NA	Vaginal	NA	37	NA	NA	Self-limited
4 (15)	Sultan	1984	29	NA	300	Hematuriaecchymoses	NA	APCC	cesarean section	NA	NA	NA	NA	NA
5 (22)	Vicente	1987	29	NA	175	NA	NA	NA	NA	NA	NA	Detected	NA	NA
6 (17)	Ries	1995	32	1	26	NA	NA	NA	Vaginal	Vaginal bleeding	1	15.2	Intracranial hemorrhage	NA
7 (14)	Solymoss	1998	34	6	14.4	NA	Prednisone	NA	NA	NA	NA	NA	NA	Self-limited
8 (23)	Coller	2004	35	2.8	885	NA	NA	NA	NA	NA	NA	NA	NA	Remission after treatment
9 (10)	Rose	2007	17	24	1.7	Mucosal bleeding	NA	NA	Vaginal	NA	NA	NA	NA	Self-limited
10 (11)	Chaari	2012	31	18	2.3	Ecchymoses	Prednisone	rFVIIa	Vaginal	Vaginal bleeding	NA	NA	NA	NA
11 (6)	Tengborn	2012	29	1	26	Ecchymoses	NA	NA	NA	NA	NA	NA	NA	NA
12 (18)	Gunel	2021	27	<1	>100	NA	NA	rFVIIa	cesarean section	NA	0.3	320	NA	NA
13 (9)	Nishi	2021	32	<1	21.6	Hematuriaecchymoses	Prednisone CsA	rFVIIa, PCC	cesarean section	heavy uterine bleeding	17.2	0.9	NA	NA

APCC, activated prothrombin complex concentrate; PCC, prothrombin complex concentrate; CsA, cyclosporin; rFVIIa, recombinant activated factor VII; NA, not applicable.

Since FVIII inhibitors are IgG immunoglobulins, they can cross the placenta and may lead to bleeding complications in newborns. Thus, neonatal management is very crucial. According to previous literature, the inhibitors may resolve spontaneously without hemorrhagic events (9). However, severe intracranial hemorrhage in a newborn infant with transplacental transfer of FVIII inhibitors was also reported, and the hemostasis was achieved by transfusion of large doses of FVIII (17). Prophylactic treatment with rFVIIa (90 μg/kg/d) was once administered to a newborn with a FVIII inhibitor titer as high as 320 BU/mL at birth, and no bleeding events occurred (18). In our case, the ultrasound screening showed no bleeding signs and we observed a spontaneous eradication of inhibitors within 2 weeks without treatment (Figure 2).

**Figure 2:**
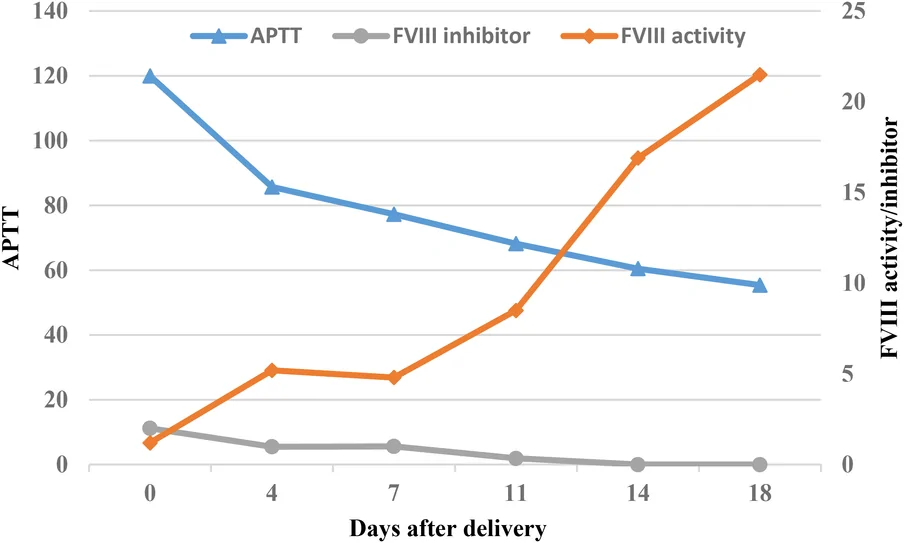
Dynamic changes of coagulatory parameters in the neonate. APTT, Activated Partial Thromboplastin Time.

## Conclusion

In conclusion, we reported successful peripartum management in an AHA primigravida who suffered an early relapse of the disease. Bypassing agents are safe, effective choices to prevent severe postpartum hemorrhage. Though FVIII inhibitors were detected in the infant, no bleeding complication occurred under close observation. It is to be noted that immunosuppressive therapies during pregnancy and after delivery failed to eradicate FVIII inhibitors, highlighting the importance of thorough pre-conception counselling for AHA patients without complete disease remission.

## Data Availability

The original contributions presented in the study are included in the article/Supplementary Material, further inquiries can be directed to the corresponding author.
